# IMOP: randomised placebo controlled trial of outpatient cervical ripening with isosorbide mononitrate (IMN) prior to induction of labour – clinical trial with analyses of efficacy, cost effectiveness and acceptability

**DOI:** 10.1186/1471-2393-6-25

**Published:** 2006-07-25

**Authors:** Shrikant Bollapragada, Fiona Mackenzie, John Norrie, Stavros Petrou, Margaret Reid, Ian Greer, Inass Osman, Jane E Norman

**Affiliations:** 1 Division of Developmental Medicine, Glasgow Royal Infirmary, Queen Elizabeth Building, 10 Alexandra Parade, Glasgow, G31 2ER, UK; 2 Princess Royal Maternity Hospital, Glasgow Royal Infirmary, 10 Alexandra Parade, Glasgow, G31 2ER, UK; 3 Centre for Healthcare Randomised Trials (CHaRT), Health Services Research Unit, Aberdeen University, Applicant and Trial Statistician CHaRT, Polwarth Building, Foresterhill, Aberdeen AB25 2ZD, UK; 4 National Perinatal Epidemiology Unit, Institute of Health Sciences, Old Road Headington, Oxford OX3 7LF, UK; 5 Public Health, University of Glasgow, Public Health and Health Policy, Division of Community Based Sciences, University of Glasgow, 1 Lilybank Gardens, Glasgow, G12 8RZ, UK

## Abstract

**Background:**

There is increasing interest in carrying out pre-induction cervical ripening on an outpatient basis. However, there are concerns about the use of prostaglandins, the agents commonly used in hospital settings for this indication, because prostaglandins induce uterine contractions that may lead to fetal hypoxia. Indeed, in a recent study we demonstrated abnormalities in 9% of fetal heart rate tracings performed following prostaglandin induced cervical ripening at term. In contrast, we confirmed in the same study that isosorbide mononitrate (IMN) (administered on an inpatient basis) was both effective in inducing cervical ripening at term, and was associated with no associated fetal heart rate abnormalities.

**Methods/design:**

The aim of this study is to determine whether IMN self administered by women on an outpatient basis improves the process of induction of labour. Specifically, we hypothesise that the use of outpatient IMN will result in a shorter inpatient stay before delivery, decreased costs to the health service and greater maternal satisfaction with ripening and induction of labour, compared with placebo treatment.

In the study described here (the "IMOP" study), women scheduled for induction of labour at term, and who require pre-induction cervical ripening will be randomised to self-administer at home either IMN 40 mg, or a placebo, each vaginally, at 48 hours, 32 hours and 16 hours before scheduled hospital admission.

After admission to hospital, treatment will revert to the usual induction of labour protocol. We will compare the primary outcomes of the elapsed time interval from hospital admission to vaginal delivery, the costs to the health service of induction of labour, and women's experience of induction of labour in the two groups.

**Discussion:**

This trial will provide evidence on the efficacy of outpatient IMN for pre-induction cervical ripening at term. We will study a formulation of IMN which is cheap and widely available. If the treatment is effective, acceptable to women, and cost effective, it could be implemented into obstetric practice worldwide.

**Trial registration:**

The trial has been registered on the International Standard Randomised Controlled Trial Number Register (ISRCTN) and given the registration number ISRTN39772441.

## Background

### Outpatient cervical ripening

Around 20% of pregnant women undergo induction of labour in the UK [1]. In primigravidae, the mean time taken from induction to delivery is between 15 and 20 hours, of which up to 12 hours is spent in the cervical ripening phase before labour itself starts. There is increasing interest in carrying out cervical ripening on an outpatient basis. Outpatient cervical ripening with prostaglandins is associated with significantly shorter admission to delivery times [2,3] and/or reduced health care costs [4]. Perhaps the most significant factor in favour of outpatient cervical ripening is women's endorsement: women were much more likely to report high satisfaction rates with outpatient compared with inpatient cervical ripening (56% v 39%, p < 0.008) [5]. However, there are concerns about the use of prostaglandins, the agents commonly used in hospital settings for this indication, and their use for outpatient cervical ripening has been declared unsafe by many authorities because of the potential for uterine contractions to cause fetal hypoxia which would remain undiagnosed in the outpatient environment [6,7]. In a recent study we demonstrated abnormalities in 9% of fetal heart rate tracings performed following prostaglandin induced cervical ripening at term. In contrast, we confirmed in the same study that IMN (administered on an inpatient basis) was both effective in inducing cervical ripening at term, with no associated fetal heart rate abnormalities.

### Properties of the ideal cervical ripening agent

An agent that ripened the cervix without stimulating uterine activity would be the ideal cervical ripening agent for outpatient use. We have previously demonstrated that vaginal IMN administered on an inpatient basis is an effective cervical ripening agent in the first trimester [8,9]. We have also shown that vaginal IMN, in the doses and at the gestation planned here, has no clinically significant adverse effects on either maternal or fetal haemodynamics [10]. In our recently published "PRIM" study [11] we found that IMN 40 mg was an effective cervical ripening agent at term. These findings are supported by the recent work of Ekerhovd et al, who showed that cervical resistance was reduced by 50% 4 hours after treatment with 40 mg vaginal IMN at term [12]. In contrast to prostaglandins, nitric oxide donors inhibit rather than stimulate uterine contractions, and promote rather than restrict uterine blood flow. Thus nitric oxide donors such as IMN appear to be the ideal cervical ripening agent for outpatient use.

### Why is a trial needed now?

To our knowledge, there are no systematic reviews on outpatient induction of labour. A Medline search using the headings "outpatient" and "cervical ripening" and restricted to studies in the English language on human subjects generated 22 publications, of which 17 are relevant to this application [2-4,6,7,13-26]. A search of the Cochrane Controlled Trials Register using the same key words did not reveal any additional studies. The majority of published studies on outpatient cervical ripening have used prostaglandins as the active agent. One has examined the use of Dilapan [26] and one a transcervical Foley catheter [24].

We found no systematic reviews or studies investigating the use of IMN for outpatient cervical ripening. Two studies have reported on the clinical use of nitric oxide donors for cervical ripening at term on an inpatient basis. In the first, 500 μg of the nitric oxide donor glyceryl trinitrate was associated with fewer episodes of uterine tachysystole compared with 3 mg PGE_2 _[27]. In the second 40 mg IMN (up to three doses at 6 hour intervals) was associated with fewer adverse events overall (including tachysystole) compared with 50 μg of the prostaglandin misoprostol (up to three doses at 6 hours intervals) [28]. In both of these studies, whilst the nitric oxide donor was considered effective, it was less effective than the comparator prostaglandin on an inpatient basis.

There is increasing interest in outpatient cervical ripening. This interest is driven at least in part by financial costs associated with an inpatient stay in the labour ward. The estimated cost for 24 hour labour ward stay is £500 – £1000 [North Glasgow University Hospitals Trust Finance department – personal communication], thus a 30% reduction in the inpatient ripening phase of 12 hours would be associated with a cost saving of £75 – £150 per patient. Additionally, there is a wish to "deinstitutionalise" the process of labour, and, where appropriate, to offer women the opportunity to remain as an outpatient for a longer period of time. Hofmeyer notes that information on women's views on cervical ripening and induction of labour are conspicuously lacking [29]. A small number of studies, including Biem et al (2003) [5] and PRIM [11] have provided some pilot evidence that women report positive views about outpatient cervical ripening. Additionally, self administration of vaginal tablets on an outpatient basis is acceptable to women in other clinical situations [30]. There is as yet no research evidence on the views of women who have experienced IMN induced cervical ripening in an outpatient setting, and women's acceptance of the novel situation of "pre-induction cervical ripening induction at home" is vital to its ultimate success. IMOP will address not only the clinical efficacy of outpatient administration of IMN for pre-induction cervical ripening, but also the economics of this strategy together with an assessment of women's views and experiences.

We believe that IMN may be the most appropriate pre-induction cervical ripening agent for outpatient use, since its uterine relaxant properties (in contrast to the uterine stimulant effects of prostaglandins) obviate the main reason for continuous fetal heart rate monitoring. A trial of outpatient IMN is particularly timely, because we have recently demonstrated the efficacy, safety and patient acceptability of inpatient cervical ripening with IMN in the PRIM study. In the PRIM study cervical ripening with IMN (two doses of 40 mg 16 hours apart) was compared on an inpatient basis with the agent conventionally used in the Princess Royal Maternity, Glasgow (prostaglandin gel, two doses of 2 mg, 16 hours apart). Briefly, the use of IMN as a cervical ripening agent was significantly more acceptable to women than the prostaglandin used as an alternative (mean [SD] visual analogue score of maternal satisfaction with treatment of 7.03 [2.71] with IMN compared with 5.79 [3.07] with prostaglandin, p < 0.0001). We also confirmed that inpatient treatment with IMN was not associated with events that would preclude outpatient use. The PRIM study evaluated the inpatient use of IMN for cervical ripening. However, we believe that it is as outpatient therapy where the effects of IMN may be the most significant advance in cervical ripening. The "IMOP" study described here will be the first to evaluate the use of IMN in this setting. We consider that it is particularly timely to embark on such a trial now, given that data from the PRIM study suggests that outpatient use of IMN is likely to be safe and effective.

## Methods and trial design

### Aims of the trial

The aim of this trial is to test the hypothesis that IMN, 40 mg every 16 hours to a total of three doses improves the process of induction of labour. Specifically we aim to test the hypotheses that outpatient pre-induction cervical ripening with IMN:

i. Reduces the elapsed time from hospital admission to delivery

ii. Reduces the costs of induction of labour

iii. Improves women's experience of induction of labour

iv. Does not increase operative delivery rates

Women scheduled for induction of labour at term, and who require pre-induction cervical ripening will be invited to participate in the study. Participants will be randomised into an active (IMN) or a control (placebo) group and asked to self-administer either IMN 40 mg, or a placebo, each vaginally, at 48 hours, 32 hours and 16 hours before scheduled admission. After admission to hospital, treatment will revert to the usual induction of labour protocol whereby prostaglandins are given until cervical ripening is achieved, and fetal membrane rupture is performed thereafter, followed by syntocinon if contractions do not start spontaneously. Data on the outcome measures listed below will be collected and compared in the two groups.

### Trial design

IMOP is a double blind, randomised, placebo controlled trial in a single clinical setting. Active and placebo treatment will be allocated in a 1:1 ratio. The primary outcome measures are the effect of outpatient pre-induction cervical ripening with IMN on:

i. The elapsed time from hospital admission to delivery (defined as the time from admission to inpatient induction or admission in labour to delivery – either vaginally or by caeserean section)

ii. The costs of induction of labour

iii. Women's experience of induction of labour (maternal satisfaction)

iv. Operative delivery rates

### Setting

The study will be carried out in a large UK NHS teaching hospital (Princess Royal Maternity, Glasgow).

### Participants

#### Inclusion criteria

Women scheduled for induction of labour will be invited to participate in the study. The rationale for induction of labour in the majority (but not all) of these women will be post-dates pregnancy. The hospital protocol is to offer induction of labour after 40 weeks and 10 days gestation, and in the PRIM study, "post dates" was the principal reason for induction of labour in over 70% of subjects. The other inclusion criteria will be as follows:

▪ Bishop Score ≤ 6

▪ Singleton pregnancy

▪ Nulliparity

▪ ≥37 completed weeks of gestation

▪ Willing to self administer vaginal tablets

#### Exclusion criteria

▪ fetal compromise of sufficient severity that daily fetal monitoring is scheduled.

### Recruitment and consent

Subjects will not be formally recruited to the study until the decision to induce labour is made. Potential subjects will be informed about the trial and given written information in the third trimester. Those women who wish to participate and who understand the nature of the trial will be asked to complete and sign two copies of the consent form. Those women who do not wish to participate, or who are ineligible will be asked (verbally) for permission to record some basic data so that the generalisability of the trial can be subsequently assessed.

Women scheduled for induction of labour at term will be recruited and randomised at an antenatal visit by the clinical research fellow, up to seven days prior to scheduled admission for induction of labour. A vaginal examination will not be repeated if one has been done and each of the components of the Bishop score documented within 7 days of anticipated start of outpatient treatment. Women willing to participate in the study will be given their randomised study medication (either IMN tablets or placebo) at this visit, with instructions to self-administer the tablets vaginally starting 48 hours prior to the scheduled time of admission for induction of labour.

### Randomisation

Randomisation to IMN or placebo will be in the ratio 1:1, using randomised permuted blocks. The randomisation schedule will be generated by the Study Data Centre at the Centre for Healthcare Randomised Trials (CHaRT) at Aberdeen University. The randomisation schedule will be used by the Clinical Trials section of the pharmacy at the Western Infirmary, Glasgow, to make up treatment packs (either active or placebo) for each patient, each labelled with the relevant unique study number. Active and placebo treatments will otherwise appear identical, so that this will be a double blind study. A copy of the randomisation schedule will be held at the Clinical Trials Pharmacy at the Western Infirmary, and at CHaRT only. None of the investigators will have access to the schedule until completion of the study.

Within one hour after recruitment of each subject, the pack number for the subject will be allocated by phone call to the automated telephone randomisation service located at CHaRT in Aberdeen.

### Cessation of randomised treatment

The following will be indications for early cessation of treatment

• Admission in labour

• Fetal membrane rupture

• Bleeding

The general practitioner and referring obstetrician will be informed. The clinical research fellow will still collect outcome data from women who have stopped taking their study medication, unless the participant asks that her data are not collected. Women who are admitted in labour, women who experience spontaneous membrane rupture and those who experience antepartum haemorrhage will be asked not to take any more doses of study drug, but will still be followed up. Women in whom fetal membranes rupture after taking study medication will be asked to come into the hospital for assessment. They will normally be offered the option of immediate augmentation with syntocinon, or delaying augmentation with syntocinon until the next morning, depending on their preference.

In the event of a participant deciding to stop their medication themselves, or if cessation is recommended by the supervising obstetrician, the clinical research fellow will complete the withdrawal CRF. The clinical research fellow will also inform the hospital pharmacy, and return any unused medication. The general practitioner and referring obstetrician will be informed.

### Interventions

The intervention is one tablet inserted vaginally on three occasions every 16 hours. In the experimental (IMN) group, a 40 mg IMN tablet will be self-administered vaginally by the patient at home 48, 32 and 16 hours before scheduled admission for induction of labour. In the control group, a placebo tablet, with an identical appearance to that of IMN, will be self-administered vaginally by the patient at home 48, 32 and 16 hours before scheduled admission for induction of labour.

#### Rationale for dosage

IMN will be given in a dose of 40 mg. Previous studies have shown that this dose is effective in inducing cervical ripening both in the first trimester [8,9] and at term [11] (PRIM) with no clinically significant effects on maternal haemodynamics [10] and without unacceptable side effects (PRIM) [11].

#### Rationale for dosage intervals

We have opted for a 16 hour dosage interval. Our previous studies suggest that systemic absorption of IMN from vaginally administered tablets is slow [31], thus a short dosage interval might lead to accumulating serum levels of drug. In the PRIM study, we gave a repeated dose of IMN after 16 hours. Haemodynamic and symptomatic side effects after the second dose of IMN were, if anything, less than after the first, suggesting that a 16 hour treatment interval is not associated with unacceptable systemic levels of drug.

#### Rationale for duration of treatment

We have opted to give IMN for 48 hours prior to admission. We anticipate that the ripening effects of IMN will continue after the first 24 hours, so that the cervix might be fully ripened by 48 hours after treatment.

With the exception of trial medication, subjects will normally be managed according to the induction of labour protocol. Thus, once a subject is admitted after trial medication, she will be managed according to the normal protocol, ignoring the use of the trial medication.

### Outcomes

Data on the primary and secondary outcomes listed below will be recorded.

#### Primary

i. Elapsed time interval from hospital admission to delivery (defined as the time from admission for inpatient induction or admission in labour to delivery – either vaginally or by caesarean section)

ii. Costs to the health service of induction of labour

iii. Women's experience of induction of labour

#### Secondary

iv. Primary clinical outcome (elapsed time from admission to delivery) for the subgroup of women who deliver vaginally

v. Operative delivery rates

vi. Incidence of unscheduled admission for reasons other than labour commencing

vii. Duration and frequency of neonatal admissions to special care

viii. Incidence of adverse maternal and fetal outcomes such as uterine hypercontractility, postpartum haemorrhage (maternal outcomes) and meconium stained liquor, five minute Apgar of less than seven (fetal outcomes)

ix. Length of labour

x. Oxytocin augmentation rates

xi. Epidural usage

xii. Proportion with unfavourable cervix at 24 hours after admission

xiii. Requirement for additional inpatient cervical ripening agent

## Collection of outcome data

### Clinical data

Outcomes i, and iv – xiii will be recorded by the attending midwifery staff and the clinical research fellow onto a dedicated Case Report Form which will be placed in the case notes of each participating subject. Data will be collected on the health service resources used in the treatment of each woman and infant during the period between randomisation and hospital discharge to determine outcome ii. The trial data collection forms will record the duration and intensity of maternal and neonatal care, based on standard criteria for level of care, as well as profile maternal and neonatal complications. The start of labour will be defined as the start of regular uterine contractions 3 in 10 (mild to moderate in intensity), with a cervical dilation equal to or greater than 3 cm. Compliance will be assessed, as far as possible, by asking subjects to complete a diary card indicating when they took study medication.

### Collection of health cost data

Observational research will also be required at the Princess Royal Maternity Hospital, to provide details of the resources and staff inputs required for the induction of labour, as well as staff time, tests, procedures, drugs and equipment entailed by complications. Tick charts will be completed by health professionals caring for all women and infants in the trial, to document the key resource and staff inputs not recorded by the trial data collection forms. A similar approach to documenting health service resource inputs has successfully been applied to other National Perinatal Epidemiology Unit economic evaluations conducted alongside clinical trials [32].

Unit costs for each resource item will be calculated by sending the finance department at the Princess Royal Maternity Hospital a detailed questionnaire requesting cost data for the main resource categories of drugs, disposables, consumables, surgical procedures, equipment, staff and overheads, and then apportioning these to different categories of patient using a 'top-down' methodology. The finance department will be visited on several occasions in order to validate the cost data provided. If required, additional cost data will be collected from secondary sources. These will be obtained towards the end of the trial period to ensure that the costs are as current as possible for the final analysis.

### Collection of maternal satisfaction/acceptability data

Outcome iii, women's experience of induction of labour, will be drawn from two approaches. First, all women will be asked to complete a short questionnaire one hour before taking IMN using simple visual analogue scales for pain and anxiety. Baseline anxiety status will be assessed using the Eysenck anxiety scale. Women will be asked to note other symptoms and comments. We will ask women to complete further questionnaires after each 16 hour treatment period. Women will be given a stamped addressed envelope to send the completed forms to the social science researcher after delivery. All women will also be asked to complete a short follow-up questionnaire after delivery to ascertain satisfaction with cervical ripening, labour and delivery, pain scores and comments on the labour (e.g. duration). Women will be given the post delivery questionnaire at the hospital for completion, and asked to hand it in prior to discharge from hospital. In addition to the questionnaire strategies described above, a consecutive series of women recruited in the second year of the study will be approached for consent to being interviewed three weeks after delivery. We aim to interview 20 women in total. The interview will collect in-depth responses to the situation of women being at home during cervical ripening, including views on whether they felt in control of the situation, how easy it was to administer the tablet, whether they managed to administer the tablet at the prescribed intervals and any other positive or negative feelings.

### Collection of health professionals attitudinal data

As it is anticipated that cervical ripening at home may change the workload within the labour suites, midwives working in the Unit will be asked to take part in focus groups. (The use of focus groups have previously been found to be valuable in studying organisational change [33]). It is anticipated that around five focus groups, each of six midwives and lasting one hour will be needed. Staff will be recruited from those working in the obstetric unit at the PRM. Those expressing interest will be given an information sheet about the study and asked to complete a consent form. The topic guide for the focus group will be generated by the social science researcher. The purpose of the focus groups will be to discuss any perceived changes in ward throughput, management, and organisation. The focus groups will also ascertain whether staff perceive there are any benefits or disadvantages to women having outpatient pre-induction cervical ripening, and explore any other topics they believe to be important in relation to this issue.

### Statistical issues

#### Sample size calculation

Three hundred women will be recruited, of whom about 150 will be in the experimental and about 150 will be in the control (placebo) group. The primary outcome on which this study is powered is the time interval from hospital admission to delivery. In women undergoing induction of labour with Bishop scores 0–4 and 5–8, the mean (SD) induction to delivery intervals are 19.65 (10.75) hours and 15.17 (6.80) hours respectively (J Norman, data on file). With 150 women in each of the IMN and placebo groups (300 in total) the study has 96% power at a 5% level of significance to detect a difference in mean time from admission to delivery of 4 hours, assuming a common standard deviation of 9 hours, using a two sample two sided t-test.

Women's experience of induction of labour will be explored qualitatively and quantitatively. As in the PRIM study, we will ask women to indicate satisfaction with the ripening process on a visual analogue scale. Assuming similar results in the PRIM study (that those pretreated with IMN will give a mean (SD) score of satisfaction of 7.0 and those who have prostaglandins only as the active ripening agent will give a mean (SD) score of satisfaction of 5.8, with a common standard deviation of 3.0, the study has 93% power to detect a significant difference in satisfaction rates, given a sample size of 150 in each group. Statistical power of 90% or above (at the 5% significance level) is maintained if the number of evaluable patients in each group is 133 or greater.

### Loss to follow up

We do not anticipate any appreciable loss to follow up i.e. women failing to return usable data on the primary outcome.

### Failure to initiate on randomised study medication

Those women who go into spontaneous labour after recruitment, but before starting trial drug, and those women who withdraw from the study at this stage will (if they consent to continuing use of their data) be included in the primary analysis, but excluded from a subsequent secondary analysis.

Since the treatment is self administered, there may be sub-optimal delivery of the assigned treatment, which may lead to a dilution of the anticipated treatment effect. For example, if 1 in 8 (or 12.5%) of the IMN women failed to properly administer the dose, the 4 hour treatment difference would be reduced to 3 hrs and 30 minutes. The study would still have 93% power to detect such a difference, and 81% power if this proportion of women rose to 1 in 4 (or 25%).

## Statistical analysis ground rules

The primary endpoint of time from admission to delivery will be compared between the two groups using a two sample two sided t-test, and then adjusted for covariates that are pre-specified as important using a linear model. The analysis will be performed at the conclusion of the study. Analysis of secondary outcomes will be using t-tests (or Wilcoxon tests) or chi-squared tests as appropriate to the distribution of the data, again with adjustment for covariates using appropriate generalised linear models.

All statistical analyses will be fully specified in a Statistical Analysis Plan, which will be signed off before the treatment codes are broken. All primary analyses will be according to the intention to treat principle. No interim analyses will be performed, other than those requested by the Data Monitoring committee. The results of these interim analyses, and/or any other unblinded data will not be revealed to the investigators or the Trial Steering Committee until after the study is complete.

Analysis of the questionnaire data will be carried out using SPSS and analysis of variance. Interviews and focus groups will be coded and analysed using a computer package (ATLAS Ti) used to facilitate analysis of qualitative data for emergence of salient themes.

A prospective economic evaluation will be conducted alongside the randomised controlled trial to estimate the cost-effectiveness of outpatient cervical ripening with IMN prior to induction of labour. The cost differences between the two groups, the IMN and placebo groups, will be measured, valued and combined with the clinical effectiveness data from the trial. The economic evaluation will incorporate data from all subjects recruited into the trial. The valuation of all resource inputs will be consistent with the notion of opportunity cost.

The economic evaluation will be conducted from a health service perspective and will take two forms; (i) an overall cost analysis and (ii) an incremental cost-effectiveness analysis, which will be expressed in several forms, for example, incremental cost per operative delivery prevented [34]. All analyses will be conducted on the basis of intention to treat. Outcomes and costs for the experimental and control groups will be compared using relative risks and 95% confidence intervals for dichotomous variables, Mann-Whitney-U tests for ordinal variables, and Student's t-tests for interval variables where parametric assumptions are met. Fieller's theorem will be used to calculate 95% confidence intervals for incremental cost-effectiveness ratios [35]. In the absence of stochastic data for all variables, a series of multi-way sensitivity analyses will be undertaken to explore the implications of uncertainty on the base-case incremental cost-effectiveness ratios. In addition, cost-effectiveness acceptability curves will be constructed using the net benefits approach [36]. All analyses will be performed with a microcomputer using Statistical Package for the Social Sciences (SPSS) software, version 11.5.

## Confidentiality and data security

Once a participant has agreed to join the trial, the clinical research fellow will record, on a standard form, identifying and contact information to be kept locally.

• Full name, address, telephone number (home and mobile)

• Date of birth

• NHS hospital number and CHI number (if available)

• General practitioner and hospital consultant name and address

• Unique trial number (to be issued by CHaRT after recruitment)

This information will not be sent to the trial office, but will be kept locally (in a locked cabinet) by the clinical research fellow as a linkage between the trial number and the patient's identity. The following information will be sent to the trial office as participant identifiers.

• Initials, date of birth, hospital number, Bishop score

• Unique trial number (to be issued by CHART)

The patient's case notes will be regarded as source data/documents. The local investigators (grantholders and clinical research fellow appointed to the study) will have access to the source data and will extract the data required for the CRF. A trial manager from CHaRT (Centre for Healthcare Randomised Trials) will also access source data to ensure quality control. No patient identifying information will leave the hospital.

The clinical research fellow will enter study CRFs onto the study database held by CHaRT in Aberdeen within one month of subject completion of the trial (defined as discharge of mother and baby from hospital) via the IMOP study web portal. CHART staff will work closely with the clinical research fellow to secure as complete and accurate data as possible. Extensive range and consistency check will further enhance the quality of the data.

CHaRT will make site visits to the study centre (PRM) to ensure quality control and quality assurance. A random sample of patient records will be reviewed to ensure accurate transcription onto the CRFs. CHaRT will review incoming CRFs and ask the clinical research fellow to complete missing data, and to clarify anomalies.

### Ethics and other regulatory issues

The study has MREC approval from the West Glasgow Ethics Committee 1 (04/S0703/35) (see additional file no 1) and LREC approval from Glasgow Royal Infirmary/Princess Royal Maternity. The MREC, will be informed of amendments to the protocol or patient information. The LREC will also be informed of these amendments at MREC's request. The study had a DDX exemption from the MHRA (13608), which was subsequently "rolled over" into a CTA. The study has been registered on the EUDRAct database 2004-000995-15.

### Data Monitoring Committee

A Data Monitoring Committee (DMC) will be established. This will consist of two obstetricians and a statistician and will be independent of the trial organisers. The DMC will advise the Steering Committee if, in its view, one or more of the randomised comparisons in the trial has provided both (a) proof beyond reasonable doubt that for all or some types of patients one particular type of intervention is clearly indicated in terms of a reduction in the induction to delivery interval, (or clearly contraindicated because of a net increase in induction to delivery interval or other adverse events), and (b) evidence that might reasonably be expected to influence materially the care of women having induction of labour by clinicians who know the results of this and comparable trials. The Steering Committee will then decide whether or not to modify intake to the trial. Unless this happens, however, the steering committee, clinical collaborators, and trial office staff (except those who supply the confidential analyses) will remain ignorant of the interim results. (Note that appropriate criteria for proof beyond reasonable doubt cannot be specified precisely. A difference of at least three standard deviations in the interim analysis of a major endpoint may be needed to justify halting, or modifying, such a study prematurely. If this criterion were to be adopted, it would have the practical advantage that the exact number of interim analyses would be of little importance, and so no fixed schedule is proposed) [37].

Interim analysis of some of the clinical outcomes (e.g. caesarean section rate, admission to hospital during ripening phase for reasons other than onset of labour) will be performed by the Data Monitoring Committee after about 180 women have been recruited. Criteria for advising the early termination of the study for safety reasons or for overwhelming evidence of benefit will be determined before any unblinded data is seen, and documented in the Minutes of the meeting of the DMC. There are no plans to stop the study early for futility. Assuming the termination criteria are not satisfied, the results will not be revealed to the investigators or participants, and no further analyses will be performed until data acquisition is complete.

### Funding and sponsorship

The study is funded by a research grant from the Charity Wellbeing at the Royal College of Obstetricians and Gynaecologists (see additional file 2). The study "sponsors" (as defined by the EU directive) are Glasgow University and North Glasgow University Hospital Division. The funder of the study has had no input into protocol design.

### Study website

A fuller version of the study protocol is available at the study website [38].

## Discussion

We believe that outpatient IMN will reduce the time from hospital admission to delivery, reduce the costs of induction of labour and improve women's experience of induction of labour (with no increase in operative delivery rates). This trial will determine whether these hypotheses are correct. If so, we anticipate that women undergoing induction of labour in the UK and in other countries worldwide could use outpatient IMN as soon as the finding are published, since the formulation of IMN we plan to investigate in this study is cheap and widely available.

## Abbreviations

IMN – Isosorbide mononitrate

## Competing interests

The author(s) declare that they have no competing interests.

## Authors' contributions

FM, JN, SP, MR, IAG, IO, and JEN were all responsible for the conception and design of the study. SB, FM, JN, SP, MR, IAG, IO, and JEN were all responsible for drafting the protocol, and are involved in the development and implementation of the study. All authors have read and given approval of the final manuscript.

## Pre-publication history

The pre-publication history for this paper can be accessed here:


